# β_1/2_ or M_2/3_ Receptors Are Required for Different Gastrointestinal Motility Responses Induced by Acupuncture at Heterotopic or Homotopic Acupoints

**DOI:** 10.1371/journal.pone.0168200

**Published:** 2016-12-15

**Authors:** Xinyan Gao, Yuxue Zhao, Yangshuai Su, Kun Liu, Xiaochun Yu, Changxiang Cui, Zhaokun Yang, Hong Shi, Xianghong Jing, Bing Zhu

**Affiliations:** 1 Institute of Acupuncture and Moxibustion, China Academy of Chinese Medical Sciences, Beijing, China; 2 People’s Hospital of Ri Zhao, Rizhao, China; National Institute for Agronomic Research, FRANCE

## Abstract

Acupuncture at homotopic acupoints or heterotopic acupoints is known to either inhibit or facilitate gastrointestinal motility, depending on the acupoint location. However, little effort has been made to investigate the roles of specific receptors (such as adrenergic and muscarinic acetylcholine receptors) in mediating the effects of acupuncture at heterotopic and homotopic acupoints. Different adrenergic receptor subtypes or cholinergic receptor subtypes are predominantly expressed in various sections of the gut, resulting in variations between the effects of acupuncture at heterotopic or homotopic acupoints on gastrointestinal motility. Here, we investigated the role of β_1_/β_2_ receptors and M_2_/M_3_ receptors in gastrointestinal motility regulated by acupuncture at ST37, a heterotopic acupoint, and ST25, a homotopic acupoint, by simultaneously recording intraluminal pressures in the distal colon and stomach or jejunum and examining fecal phenol red excretion in β_1/2_ receptor-knockout mice and M_2/3_ receptor-knockout mice. We found that knockout of the M_2/3_ receptor significantly inhibited ST37 acupuncture-induced enhancement of gastric motility, jejunal motility, and colonic motility. Additionally, knocking out of the β_1/2_ receptor significantly diminished the ST25 acupuncture-induced inhibition of gastric motility and jejunal motility without significantly altering the enhancement of colonic motility induced by acupuncture at ST25. Acupuncture at ST37 significantly accelerated gastrointestinal transition in β_1/2_ receptor-knockout mice and their wild-type littermates. However, this acceleration of gastrointestinal transition was markedly diminished in M_2/3_ receptor-knockout mice relative to their wild-type littermates. Acupuncture at ST25 significantly increased gastrointestinal transition in β_1/2_ receptor-knockout mice and significantly decreased gastrointestinal transition in M_2/3_ receptor-knockout mice without altering gastrointestinal transition in wild-type littermates of either. Our study revealed that M_2/3_ receptors are required for the gastrointestinal motility associated with whole gastrointestinal transition enhanced by acupuncture at heterotopic acupoints, whereas β_1/2_ receptors are required for the same gastrointestinal motility processes inhibited by acupuncture at homotopic acupoints. Therefore, our findings reveal important biological mechanisms underlying acupuncture treatment of disorders involving gastrointestinal motility dysfunction.

## Introduction

The efficacy of acupuncture-like somatic stimulation on the treatment of gastrointestinal motility disorders has been established in both clinical and research settings [1–7]. Namely, the effect of acupuncture on gastric motility is primarily known to be associated with autonomic reflexes and the gut-brain axis [8]. Stimulation at homotopic acupoints, where afferent innervation occurs in the same or adjacent segment from that in which the efferents innervate visceral organs, decreases intra-gastrointestinal pressure, with or without spinalization. Conversely, acupuncture at heterotopic acupoints, involving different segmental innervation of the spinal cord to visceral organs, induces gastrointestinal facilitation only in complete spinal rats [9]. However, little effort has been made thus far to investigate the roles of specific ligand receptors (such as adrenergic and muscarinic acetylcholine receptors) in mediating the effects of acupuncture, at heterotopic or homotopic acupoints, on motility of various sections of gut.

β-adrenoceptors (β-ARs) are divided into three different subtypes (β_1–3_), all of which are widely expressed in smooth muscle organs. β-AR stimulation has been shown to consistently produce a relaxation response in a range of human gastrointestinal smooth muscle preparations. The range of muscle relaxation responses in the ileum, colon, and rectum, following β-AR agonism by various pharmacological agents, suggests differing roles for β-ARs in longitudinal and circular smooth muscle cell types [10]. β_1_-and β_2_-ARs may also have roles in gastrointestinal motility that are regulated by the sympathetic nervous system [11].

Muscarinic acetylcholine receptors are comprised of five distinct subtypes (M_1-5_), and are widely distributed in smooth muscle organs throughout the body, including the gastrointestinal tract [12–14]. In gastrointestinal smooth muscle tissues, M_2_ and M_3_ receptors are preferentially expressed [12]. Studies involving the recent utilization of mutant mouse strains lacking specific muscarinic receptor subtypes suggest that not only M_3,_ but also M_2_ receptors, play direct roles in inducing contraction in gastric and ileal smooth muscle tissues [15–19]. Taken together, previous research indicates that specific adrenergic and muscarinic acetylcholine receptor subtypes modulate specific gastrointestinal relaxation or contraction activities.

In the present study, we hypothesized that the variation in distribution of adrenergic and muscarinic acetylcholine receptor subtypes in different gastrointestinal tissues may explain the differing effects of acupuncture stimulation, at their respective heterotopic or homotopic acupoints, on gastrointestinal motility. Using β_1/2_ adrenergic receptor-knockout mice (β_1/2_-AR KO) and M_2/3_ muscarinic receptor-knockout mice (M_2/3_-R KO), we examined the specific roles of these cholinergic receptor subtypes in mediating contraction or relaxation of the stomach, jejunum, and colon, induced by acupuncture at heterotopic or homotopic acupoints, relative to their respective organs. We found that acupuncture at ST37 could enhance gastrointestinal motility involved in whole gastrointestinal transition via activating M_2/3_ receptors; whereas acupuncture at ST25 could slow down a majority of gastrointestinal motility through activating β_1/2_ receptors.

## Materials and Methods

### Animals

This study was carried out in strict accordance with the recommendations in the Guide for the Care and Use of Laboratory Animals of the National Institutes of Health. The protocol was approved by the Committee on the Ethics of Animal Experiments of China Academy of Chinese Medical Sciences (Approval Number: AE20110510-001). Male β_1/2_ adrenergic receptor-knockout mice (β_1/2_-AR KO), M_2/3_ muscarinic receptor-knockout mice (M_2/3_-R KO), and their same gender wild-type (WT) littermates (6–8 weeks) were purchased from Vital River Laboratories or Nanjing Biomedical Research Institute of Nanjing University. Genetic background of transgenic mice was shown in S1 file. Mice were housed in standard animal facilities in which the room temperature was maintained at 24 ± 2°C, humidity at 60–70%, and noise levels at lower than 60 dB. All animals were grouped into two or three with *ad libitum* access to food, which was purchased from Beijing Huafukang Biotechnology Company Limited (Lot number SCCK (Jing) 2014–0008) meeting the national standard GB 14924.3–2010, and to water from a reverse osmosis system. The bedding material and drinking water were replaced every day to keep cages clean and dry. The animals were maintained on a standard 12-hour light-dark cycle (dark cycle 8:00 PM-8:00 AM) and allowed to acclimate to the housing conditions for seven days prior to the experiment.

### Acupuncture stimulation

In this study, the acupoints ST37 (Shangjuxu) and ST25 (Tianshu) were selected. ST37, which lies 1.5 mm lateral to the anterior tubercle of the tibia and 5 mm below the knee joint, is innervated by L4 spinal afferents in the mouse. ST25, which is level with the navel and 3 mm lateral to the anterior median line, is innervated by the T10 spinal segment. As the stomach, jejunum, and colon of the mouse are innervated by the sympathetic efferents T6-10, T9-10 and L1, and L1-2, respectively, ST25 is a homotopic point to the stomach and jejunum, but a heterotopic point to the colon, whereas ST37 is a heterotopic acupoint to the stomach, jejunum, and colon. The acupuncture needles (0.25 × 25 mm, Suzhou Hwato Medical Instruments, China) were manually inserted unilaterally, vertically, or slightly obliquely to a depth of approximately 5 mm, then rotated right and left, at a frequency of 2 Hz for 60 seconds. Briefly, after the needle was inserted, at a 5-mm depth, into each acupoint, the needle body was twisted using the thumb and index finger 180° anticlockwise and then 180° clockwise 120 times in 60 seconds. Manual acupuncture stimulation will be abbreviated as MA hereafter.

### Dry-wet ratio of feces

As described previously [20, 21], the dry-wet ratio of feces was measured to determine gastrointestinal transition rate in WT, β_1/2_-AR KO, and M_2/3_-R KO mice. Mice were separately placed in cages with wire flooring insets and allowed *ad libitum* access to water and food. The feces were collected after 30 minutes. The feces from each mouse were weighed before and after dehydration in an electric drying oven (DHG-9240A, Yi Heng Science and Technology Ltd., China) at 50°C for five hours. The dry-wet ratio of the feces is expressed as: $\frac{Dry\;weight\;\left(g\right)}{Wet\;weight\;\left(g\right)}\times 100\%$.

### Surgical preparation

All mice were fasted overnight prior to surgery and electrophysiological recording. Mice were induced to a surgical plane of anesthesia with a dose of urethane (1.0–1.2 g/kg, i.p., Sigma, USA). Supplementary anesthesia (0.3 g/kg, i.p.) was administered if the limb withdrawal reflex or fluctuation in heart rate was observed in response to paw clamping. Core body temperature was monitored and maintained at 37.0 ± 0.5°C by using a feedback-controlled electric blanket (ALC-HTP, Shanghai Alcott Biotech Co., Ltd, China). The tracheae were cannulated, but not immobilized, to avoid respiratory tract congestion, and a catheter was inserted into the left jugular vein for administration of solution. Heart rate was recorded by inserting three-pin electrodes intramuscularly into the bilateral forelimbs and unilateral hindlimb. Electrodes were connected to a bridge amplifier which registered intraluminal pressure using a multichannel data acquisition system. Heart rate was continuously monitored to maintain anesthetic depth and to avoid marked cardiac fluctuations caused by drug administration. An overdose of urethane (3 g/kg, i.p.) was administered to euthanize the animals following completion of the experiment.

### Recording of gastric motility

A polyurethane tube of 1 mm-diameter, attached to an empty 3-mm-diameter latex balloon, was inserted into the stomach through the mouth and esophagus. A syringe was attached to a cannula through a T-coupling tube to inflate and deflate the balloon with warm water. The balloon was filled with 0.05–0.1 mL of warm water, producing 80–120 mm H_2_O intragastric pressure. The balloon pressure was measured by a transducer connected to an amplifier through a polyethylene tube (1.5 mm outside diameters) and recorded using a multichannel data acquisition workstation (Micro1401-3, Cambridge Electronic Design, England) or Mac Lab system (AD Instruments, Australia). Following 30 minutes of stable semi-fasted gastric motor activity, acupuncture stimulation was conducted on various acupoints in random order, and motility was recorded for an additional 30 minutes.

### Recording of jejunal motility

A sterile medial abdominal incision was made and a manometric balloon was surgically implanted into the jejunum approximately 6–8 cm downstream of the distal pylorus. The balloon was filled with approximately 0.05 mL of warm water and connected to a piece of polyethylene tubing, generating an intraluminal pressure of approximately 100 mm H_2_O. Pressure in the intestinal lumen was measured using a transducer through the polyethylene tube and recorded using the Mac Lab system. Following 30 minutes of stable jejunal motility recording, acupuncture stimulation was conducted on various acupoints in random order, and motility was recorded for an additional 30 minutes.

### Recording of distal colonic motility

In this study, we recorded gastric and colonic motilities or jejunal and colonic motilities simultaneously. In order to avoid impairment of lower gastrointestinal transmission caused by upper segment surgery, gastric and colonic recordings were noninvasive whereas jejunal recordings were inevitably invasive surgical procedures. After placing the recording balloon in the stomach or jejunum, a second manometric balloon was inserted and positioned 2–3 cm inside the anus. The balloon was connected to a piece of polyethylene tubing and filled with approximately 0.05 mL of warm water, generating an intraluminal pressure of approximately 100 mm H_2_O. Pressure in the intestinal lumen was measured using a transducer through the polyethylene tubing and recorded using the Mac Lab system. Following thirty minutes of stable colonic motility recording, acupuncture stimulation was conducted on various acupoints in random order, and motility was recorded for an additional 30 minutes.

### Measurement of gastrointestinal transition

In order to compare gastrointestinal function in β_1/2_-AR KO and M_2/3_-R KO mice and their WT littermates, a phenol red excretion test was performed as described in previous reports, with slight modification [22, 23]. Ten β_1/2_-AR KO, ten M_2/3_-R KO mice, and their respective numbers of WT littermates were utilized. Mice were fasted for 12 hours (overnight) with *ad libitum* access to water prior to the experiment. Mice were administered phenol red diluted in sterile saline via oral gavage (0.5 mg/mL at a volume of 0.02 mL/g body weight), and feces were collected for six hours. During acupuncture stimulation trials, mice were gently immobilized in small dark pockets with holes to expose their legs or abdomens. In order to minimize stress, all animals had been trained for one week prior to the phenol red experiment. Briefly, the mice were gently placed into small dark pockets, without struggling, for 10 minutes. This training was repeated once a day for one week. The needle was inserted into ST37 or ST25, through holes in the pocket, for a 10-minute period. Acupuncture was performed manually for 10 min at ST37 or ST25 at 2 Hz. Excretion of phenol red was recorded on the following day after gavage.

The fecal matter collected from each mouse was homogenized in 10 mL of 5% NaHCO_3_ solution. The suspension was centrifuged at a speed of 2,000 RPM/min for 10 minutes. The supernatant (5 mL) was harvested. The optical density (OD) of each sample was detected at 546 nm wave length using the Thermo VARIOSKAN FLASH (Thermo Scientific, USA). A standard OD/concentration curve was generated with OD values detected from the following concentrations of phenol red in 5% NaHCO_3_: 1 mg/L, 2 mg/L, 4 mg/L, 8 mg/L, and 16 mg/L.

### Data analysis

All data are displayed as means ± SE. Statistical analysis was performed using the SPSS (Statistical Package of Social Science, version 20.0 for Windows). All the measurement data were firstly analyzed by Kolmogorov-Smimov test to determine whether they accorded with normal distribution. Unpaired t test was used if the data satisfied normal distribution (Kolmogorov-Smimov test: P>0.05). The Wlicoxon Mann Whitney test was performed for assessment of parameters that were not normally distributed. P values < 0.05 were considered to represent statistical significance. Offline analysis of electrophysiological recordings was conducted using Spike2 software (Cambridge Electronic Design, England). In the present study, acupuncture was performed if stable contractive motility was observed during interperistaltic intervals, and avoided during peristalsis since its amplitude was highly fluctuated. We set an appropriate amplitude threshold for raw data analysis according to the baseline waveform for each mouse; the supraliminal waves were then selected for statistical analysis. Electrophysiological recording data are displayed as a per cent change from baseline before acupuncture stimulation.

## Results

### Basal gastrointestinal motility and fecal characteristics in β_1/2_-AR KO and M_2/3_-R KO mice

**Fig 1:**
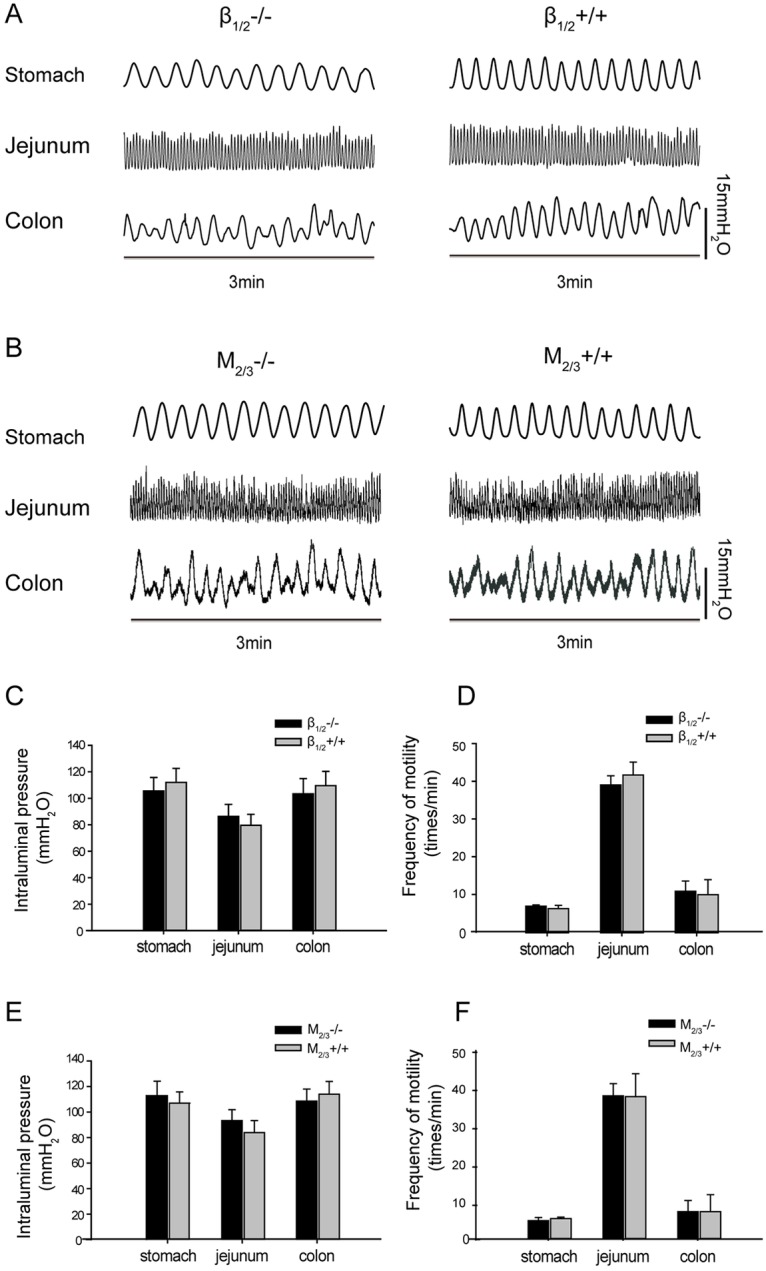
Gastric, jejunal, and distal colonic motility in β_1/2_-AR KO mice and M_2/3_-R KO mice. **(A)** Representative traces of gastric, jejunal, and distal colonic motility of β_1/2_-AR KO mice and their WT littermates **(B)** Representative traces of gastric, jejunal, and distal colonic motility of M_2/3_-R KO mice and their WT littermates **(C)** There were no significant differences in gastric, jejunal, and distal colonic motility between β_1/2_-AR KO mice and their WT littermates (unpaired *t*-test, n = 18 in each group). **(D)** There were no significant differences in the gastric, jejunal, and distal colonic motility frequencies between β_1/2_-AR KO mice and their WT littermates (unpaired *t*-test, n = 18 in each group). **(E)** There were no significant differences in gastric, jejunal, and distal colonic motilities between M_2/3_-RKO mice and their WT littermates (unpaired *t*-test, n = 18 in each group). **(F)** There were no significant differences in gastric, jejunal and distal colonic motility frequencies between M_2/3_-RKO mice and their WT littermates (unpaired *t*-test, n = 18 in each group).

Basal gastric motility, jejunal motility, and colonic motility were measured in β_1/2_-AR KO and M_2/3_-R KO mice to determine possible deviations from WT standards. As show in Fig 1A, 1C and 1D, no significant differences in basal gastrointestinal motility were observed between β_1/2_-AR KO mice and their WT littermates. For β_1/2_-AR KO mice, intragastric, intrajejunal, and intracolonic pressure and contraction frequencies were 105.49 ± 10.18 mm H_2_O, 5.70 ± 0.30 times/min; 86.31 ± 9.08 mm H_2_O, 38.57 ± 2.17 times/min; and 103.33 ± 11.58 mm H_2_O, 9.29 ± 2.45 times/min, respectively. For the WT littermates of β_1/2_-AR KO mice, intragastric, intrajejunal, and intracolonic pressures and contraction frequencies were 112.03 ± 10.51 mm H_2_O, 5.09 ± 0.79 times/min; 79.56 ± 8.32 mm H_2_O, 41.04 ± 3.06 times/min; and 109.53 ± 10.81 mm H_2_O, 8.49 ± 3.55 times/min, respectively.

As shown in Fig 1B, 1E and 1F, no significant differences were observed in basal gastrointestinal motility between M_2/3_-R KO mice and their WT littermates. For M_2/3_-R KO mice, intragastric, intrajejunal, and intracolonic pressures and contraction frequencies were 112.81 ± 11.38 mm H_2_O, 5.47 ± 0.79 times/min; 93.29 ± 8.51 mmH_2_O, 37.22 ± 3.08 times/min; and 108.51 ± 9.52 mm H_2_O, 7.77±2.75 times/min, respectively. For WT littermates of M_2/3_-R KO mice, intragastric, intrajejunal, and intracolonic pressures and contraction frequencies were 106.87 ± 8.91 mm H_2_O, 6.01 ± 0.37 times/min; 83.88 ± 9.30 mm H_2_O, 36.95 ± 5.83 times/min; and 113.93 ± 10.07 mm H_2_O, 7.78 ± 4.28 times/min, respectively.

**Fig 2:**
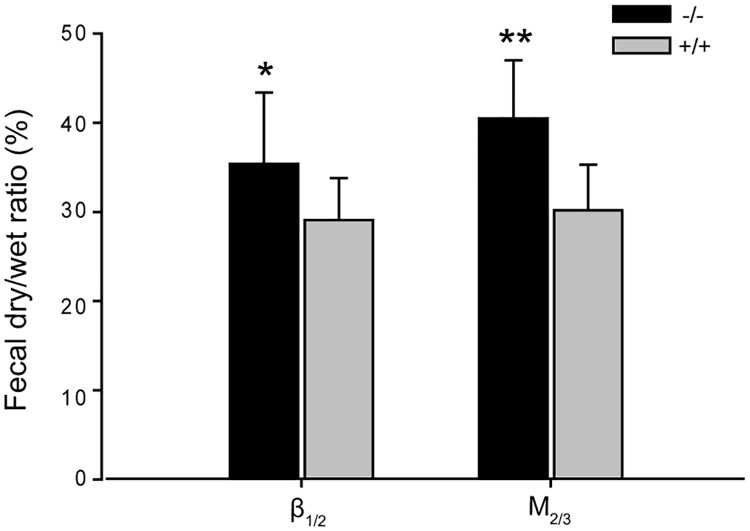
Dry-wet ratio in β_1/2_-AR KO mice and M_2/3_-R KO mice. Dry-wet ratio in both β_1/2_-AR KO mice and M_2/3_-R KO mice was significantly higher than in their WT littermates (* P < 0.05, ** P < 0.01, unpaired *t*-test, n = 18 in each group).

Fig 2 shows the fecal characteristics of β_1/2_-AR and M_2/3_-R KO mice, expressed as dry-wet ratios. The dry-wet ratio of fecal matter from β_1/2_-AR KO mice was 35.41 ± 8.01%, which was significantly higher than that of their WT littermates (29.08 ± 4.65%, P < 0.05). The dry-wet ratio of fecal matter from M_2/3_-R KO mice was 40.49 ± 6.47%, which was significantly greater than that of their WT littermates (30.16 ± 5.07%, P < 0.01). The above data suggest that the absence of β_1/2_-ARs or M_2/3_-Rs may increase water absorption without affecting basal gastric, jejunal, and colonic motilities.

### The effect of acupuncture at ST37 or ST25 on gastric motility in β_1/2_-AR KO or M_2/3_-R KO mice

**Fig 3:**
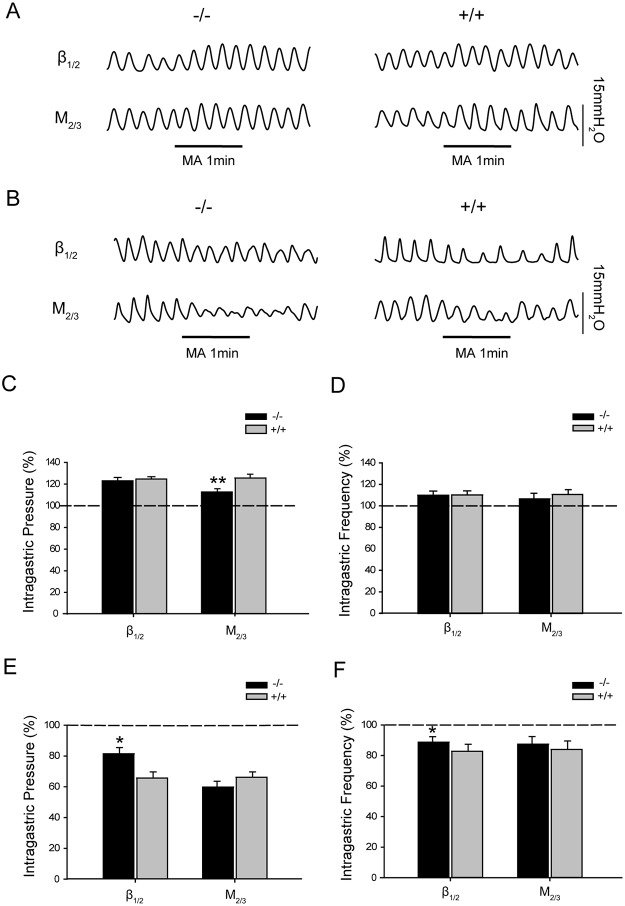
Effect of acupuncture at ST37 or ST25 on gastric motility in β_1/2_-AR KO mice and M_2/3_-R KO mice. **(A)** Representative traces of gastric motility regulated by acupuncture at ST37 in β_1/2_-AR-KO mice and M_2/3_-R-KO mice. **(B)** Representative traces of gastric motility regulated by acupuncture at ST25 in β_1/2_-AR-KO mice and M_2/3_-R-KO mice. **(C)** β_1/2_-AR deletion did not affect the enhancement of intragastric pressure by acupuncture at ST37 relative to WT littermates (unpaired *t*-test, n = 10 in each group). M_2/3_-R deletion significantly reduced the increase in intragastric pressure caused by ST37 relative to WT littermates (** P < 0.01, unpaired *t-*test, n = 10 in each group). The dashed line denotes basal intragastric pressure before acupuncture. **(D)** β_1/2_-AR deletion did not change the gastric motility frequency enhanced by acupuncture at ST37 (unpaired *t*-test, n = 10 in each group). M_2/3_-R deletion did not significantly change the increase of gastric motility frequency caused by acupuncture at ST37 relative to WT littermates (unpaired *t*-test, n = 10 in each group). The dashed line denotes basal intragastric frequency before acupuncture. **(E)** β_1/2_-AR deletion significantly changed intragastric pressure reduced by acupuncture at ST25 relative to WT littermates (* P < 0.05, unpaired *t*-test, n = 10 in each group); M_2/3_-R deletion did not significantly change the decrease in intragastric pressure caused by acupuncture at ST25 relative to WT littermates unpaired *t*-test, n = 10 in each group). The dashed line denotes basal intragastric pressure before acupuncture. **(F)** β_1/2_-AR or deletion changed gastric motility frequency induced by acupuncture at ST25 significantly relative to WT littermates (* P < 0.05, unpaired *t*-test, n = 10 in each group). M_2/3_ deletion did not significantly change gastric motility frequency induced by acupuncture at ST25 relative to WT littermates (unpaired *t*-test, n = 10 in each group). The dashed line denotes basal gastric frequency before acupuncture; MA: manual acupuncture.

In order to investigate the effects of manual acupuncture stimulation (MA) at heterotopic or homotopic acupoints, in various segments, on gastrointestinal motility in β_1/2_-AR KO or M_2/3_-R KO mice, the acupoints ST37 and ST25 were selected (for rationale, see Methods section). As shown in S1A–S1C Fig and S2 File, MA at ST37 and ST25 exhibited significant effects on gastric motility compared with sham acupuncture. Fig 3A, 3C and 3D show that MA at ST37 elicited an increase in intragastric pressure (22.56 ± 2.87%) and ingastric contraction frequency (10.59 ± 3.73%) in β_1/2_-AR KO mice, with no significant differences relative to their WT littermates (intragastric pressure: 24.23 ± 2.02%; frequency: 10.91 ± 3.57%). In M_2/3_-R KO mice, acupuncture at ST37 increased intragastric pressure (12.51 ± 2.89%), significantly less than their WT littermates (25.06 ± 3.42%, P < 0.01). However, the increase in the frequency of gastric contraction (7.41 ± 4.90%) was not significantly different from that observed in WT mice (11.25 ± 4.30%). These data suggest that knockout of M_2/3_ receptors inhibits the augmentation of gastric motility induced by acupuncture at the heterotopic acupoint ST37.

Fig 3B, 3E and 3F show that, in β_1/2_-AR KO mice, MA at ST25 decreased intragastric pressure (18.52 ± 3.99%), which represented a significant difference compared to that observed in their WT littermates (34.26 ± 4.05%, P < 0.05); the contraction frequency (11.33 ± 3.61%) was significantly different from that in WT littermates (17.28 ± 4.62%, P < 0.05). In M_2/3_-R KO mice, MA at ST25 decreased intragastric pressure (40.18 ± 3.80%) and contraction frequency (12.62 ± 4.98%), with no statistical difference observed relative to their WT littermates (intragastric pressure: 33.83 ± 3.47%; frequency: 15.95 ± 5.47%). These data suggest that β_1_/β_2_ receptors play a role in the inhibition of gastric motility induced by acupuncture at homotopic acupoints.

### Acupuncture at ST37 and ST25 induces differing effects on jejunal motility between β_1/2_-AR KO and M_2/3_-R KO mice

**Fig 4:**
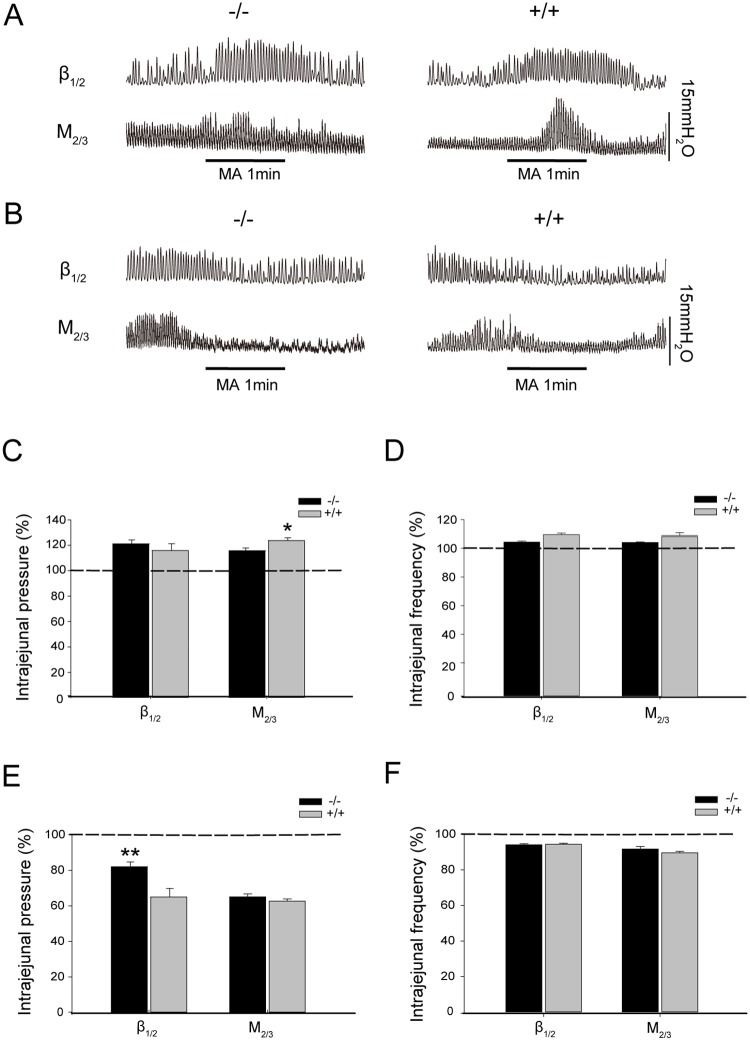
Effect of acupuncture at ST37 or ST25 on jejunal motility in β_1/2_-AR KO mice and M_2/3_-R KO mice. **(A)** Representative traces of jejunal motility regulated by acupuncture at ST37 in β_1/2_-AR KO mice and M_2/3_-R KO mice. **(B)** Representative traces of jejunal motility regulated by acupuncture at ST25 in β_1/2_-AR KO mice and M_2/3_-R KO mice. **(C)** β_1/2_-AR deletion did not change intrajejunal pressure increased by acupuncture at ST37 relative to WT littermates (unpaired *t*-test, n = 10 in each group). M_2/3_-R deletion significantly reduced the increase in intrajejunal pressure caused by acupuncture at ST37 relative to WT littermates (* P < 0.05, unpaired *t*-test, n = 10 in each group). The dashed line denotes basal intrajejunal pressure before acupuncture. **(D)** β_1/2_-AR or M_2/3_-R deletion did not change the jejunal motility frequency induced by acupuncture at ST37 relative to their WT littermates (unpaired *t*-test, n = 10 in each group). The dashed line denotes basal intrajejunal frequency before acupuncture. **(E)** β_1/2_-AR deletion significantly increased intrajejunal pressure reduced by acupuncture at ST25 relative to WT littermates (** P < 0.01, unpaired *t-*test, n = 10 in each group); M_2/3_-R deletion did not significantly affect the decrease in intrajejunal pressure caused by acupuncture at ST25 relative to WT littermates (unpaired *t*-test, n = 10 in each group). The dashed line denotes basal intrajejunal pressure before acupuncture. **(F)** β_1/2_-AR or M_2/3_-R deletion did not change jejunal motility frequency mediated by acupuncture at ST25 relative to WT littermates (unpaired *t*-test, n = 10 in each group). The dashed line denotes basal jejunal frequency before acupuncture; MA: manual acupuncture.

Previous work in our laboratory has demonstrated that acupuncture at a given acupoint may induce opposing visceral responses, depending on the homeostatic state of the individual [24, 25]. Here, we attempted to determine whether acupuncture at ST37or ST25 produces differing effects on jejunal motility. S1A, S1B and S1D Fig and S2 File showed that MA at ST37 and ST25 displayed significant effects on jejunal motility compared with sham acupuncture. As shown in Fig 4A, 4C and 4D, MA at ST37 increased intrajejunal pressure (21.96 ± 3.10%) and contraction frequency (4.32 ± 0.72%) in β_1/2_-AR KO mice, with no significant differences between these mice and their WT littermates (intrajejunal pressure: 16.69 ± 5.38%; frequency: 9.49 ± 1.08%). In M_2/3_-R KO mice, MA at ST37 increased intrajejunal pressure (16.57 ± 2.09%), with a significant difference relative to their WT littermates (24.47 ± 2.33%, P < 0.05) (Fig 4C). However, the increase in contraction frequency (6.49 ± 0.50%) was not significantly different compared with WT mice (8.38 ± 2.03%) (Fig 4D). These data suggest that M_2_/M_3_ receptors play an important role in the enhancement of jejunal motility induced by acupuncture at the heterotopic acupoint ST37.

MA at ST25 decreased intrajejunal pressure (19.21 ± 2.71%) in β_1/2_-AR KO mice, which was significantly different compared with that of their WT littermates (36.15 ± 4.77%, P < 0.01) (Fig 4B, 4E and 4F). Both β_1/2_-AR KO mice and their WT counterparts displayed a decrease in jejunal contraction frequency (β_1/2_-AR KO: 5.87 ± 0.60%; WT: 5.58 ± 0.62%); however, the differences between the two groups were not statistically significant. In M_2/3_-R KO mice, MA at ST25 decreased intrajejunal pressure (36.06 ± 1.61%) with no significant difference compared with that of their WT littermates (38.49 ± 1.23%) (Fig 4E). The change in jejunal contraction frequency in M_2/3_-R KO mice (8.33 ± 1.49%) was not significantly different from that in their WT littermates (10.38 ± 0.75%) (Fig 4F).These data suggest that β_1_/β_2_ receptors mediate the inhibition of jejunal motility induced by acupuncture at homotopic acupoints.

### Acupuncture at ST37 and ST25 induces differing effects on distal colon motility between β_1/2_-AR KO and M_2/3_-R KO mice

**Fig 5:**
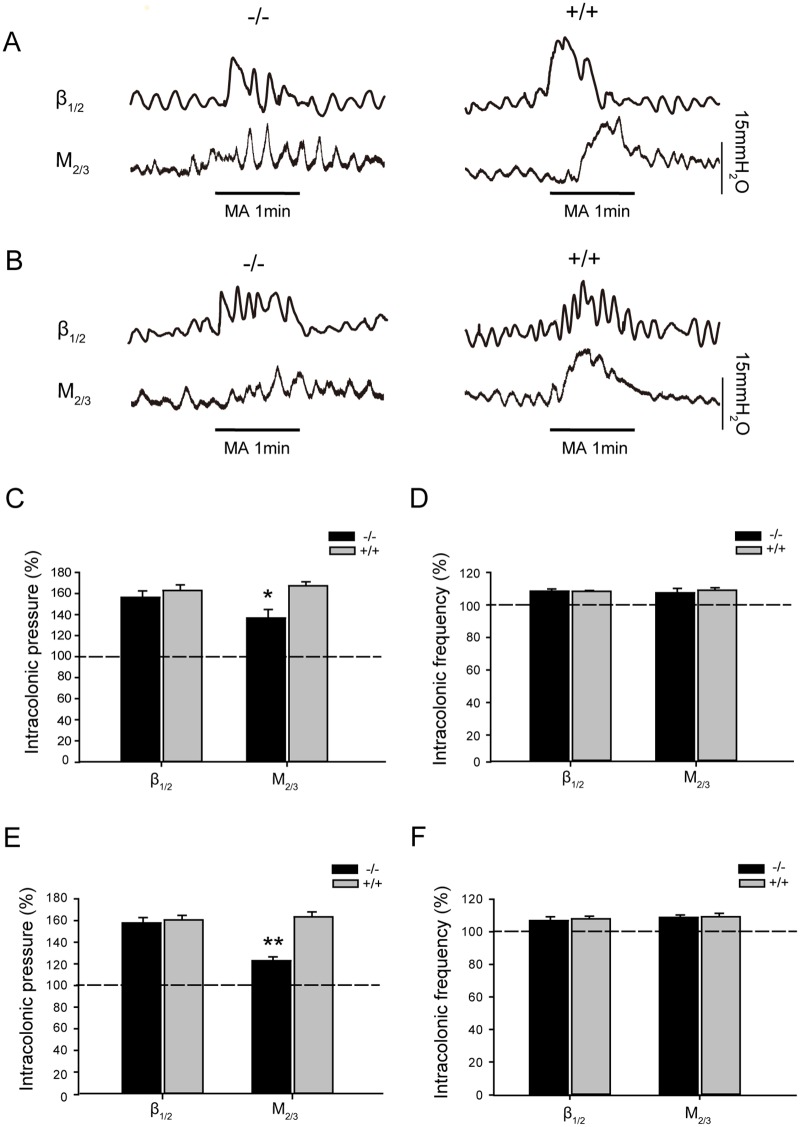
Effect of acupuncture at ST37 or ST25 on distal colonic motility in β_1/2_-AR KO mice and M_2/3_-R KO mice. **(A)** Representative traces of distal colonic motility regulated by acupuncture at ST37 in β_1/2_-AR KO mice and M_2/3_-R KO mice. **(B)** Representative traces of distal colonic motility regulated by acupuncture at ST25 in β_1/2_-AR KO mice and M_2/3_-R KO mice. **(C)** β_1/2_-AR deletion did not affect the increase in intracolonic pressure due to acupuncture at ST37, relative to WT littermates (unpaired *t*-test, n = 10 in each group). M_2/3_-R deletion significantly reduced the increase in intracolonic pressure due to acupuncture at ST37 relative to WT littermates (* P < 0.05, unpaired *t*-test, n = 10 in each group). The dashed line denotes basal intracolonic pressure before acupuncture. **(D)** β_1/2_-AR or M_2/3_-R deletion did not affect the change in distal colonic motility frequency induced by acupuncture at ST37 relative to WT littermates (unpaired *t*-test, n = 10 in each group). The dashed line denotes basal intrajejunal frequency before acupuncture. **(E)** β_1/2_-AR deletion did not significantly change the increase in intracolonic pressure due to acupuncture at ST25 relative to WT littermates (unpaired *t*-test, n = 10 in each group); M_2/3_-R deletion significantly reduced the increase in intracolonic pressure resulting from acupuncture at ST25 relative to WT littermates (** P < 0.01, unpaired *t*-test, n = 10 in each group). The dashed line denotes basal intracolonic pressure before acupuncture. **(F)** β_1/2_-AR or M_2/3_-R deletion did not change distal colonic motility frequency induced by acupuncture at ST25, relative to WT littermates (unpaired *t*-test, n = 10 in each group). The dashed line denotes basal jejunal frequency before acupuncture; MA: manual acupuncture.

As shown in S1A, S1B and S1E Fig and S2 File, MA at ST37 and ST25 displayed significant effects on distal colonic motility compared with sham acupuncture. MA at ST37 increased intracolonic pressure (56.27 ± 6.19%) and distal colon contraction frequency (8.39 ± 1.36%) in β_1/2_-AR KO mice; however, no significant differences were found relative to their WT littermates (intracolonic pressure: 62.80 ± 5.47%; frequency: 8.32 ± 0.58%) (Fig 5A, 5C and 5D). Though MA at ST37 increased intracolonic pressure in M_2/3_-R KO mice (36.66 ± 8.09%), the increase is significantly less than that of their WT littermates (67.32 ± 3.75%, *p*<0.05) (Fig 5C). The increase in M_2/3_-R KO distal colon contraction frequency (7.36 ± 2.79%) was not significantly different from that of WT mice (9.11 ± 1.47%) (Fig 5A and 5D). These data suggest that M_2_ and M_3_ receptors play an important role in the regulation of distal colonic motility regulated by acupuncture at ST37.

MA at ST25 increased intracolonic pressure (57.80 ± 4.88%) and distal colon contraction frequency (6.93 ± 2.37%) in β_1/2_-AR KO mice; however, no significant differences were found relative to their WT littermates (intracolonic pressure: 60.56 ± 4.02%; frequency: 8.02 ± 1.60%) (Fig 5B, 5E and 5F). MA at ST25 increased intracolonic pressure in M_2/3_-R KO mice (22.88 ± 3.45%), and this increase was significantly different compared to that observed in WT littermates (63.37 ± 4.62%, *p*<0.01) (Fig 5B and 5E). However, the increase in M_2/3_-R KO colon contraction frequency (8.80 ± 1.59%) was not significantly different from that of WT mice (9.27 ± 2.06%) (Fig 5B and 5F).These data suggest that M_2_ and M_3_ receptors mediate the increase of distal colonic motility induced by acupuncture at ST25.

### Effects of acupuncture at ST37 or ST25 on gastrointestinal transition

**Fig 6:**
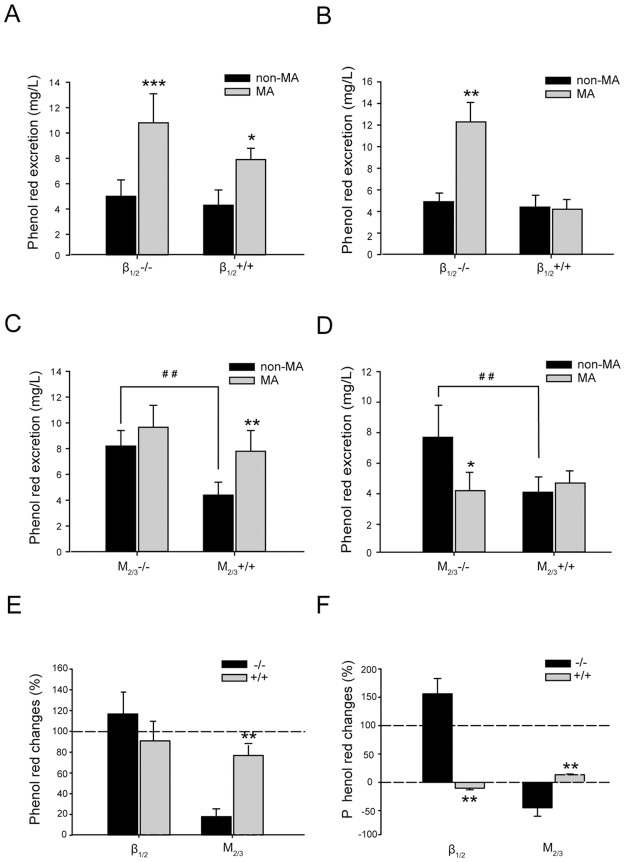
Effect of acupuncture at ST37 or ST25 on phenol red excretion in β_1/2_-AR KO mice and M_2/3_-R KO mice. **(A)** β_1/2_-AR deletion did not significantly increase phenol red excretion in feces (unpaired *t*-test, n = 10 in each group); acupuncture at ST37 increased fecal phenol red excretion significantly, relative to non-acupuncture, in both β_1/2_-AR KO mice and their WT littermates (* P < 0.05, ** P < 0.01; unpaired *t*-test, n = 10 in each group). **(B)** β_1/2_-AR deletion did not increase phenol red excretion in feces significantly (unpaired *t*-test, n = 10 in each group); Acupuncture at ST25 increased fecal phenol red excretion significantly relative to non-acupuncture in β_1/2_-AR KO mice (** P < 0.01; unpaired *t*-test, n = 10 in each group); **(C)** M_**2/3**_-R deletion increased phenol red excretion in feces significantly (## P < 0.01, unpaired *t*-test, n = 10 in each group); Acupuncture at ST37 facilitated fecal phenol red excretion significantly relative to non-acupuncture in WT littermates (** P < 0.01; unpaired *t*-test, n = 10 in each group). **(D)** M_2/3_-R deletion increased phenol red excretion in feces significantly (## P < 0.01, unpaired *t*-test, n = 10 in each group); Acupuncture at ST25 decreased fecal phenol red excretion significantly relative to non-acupuncture in M_2/3_-R KO mice (*P < 0.05; unpaired *t*-test, n = 10 in each group). **(E)** The rate of increase in phenol red excretion induced by acupuncture at ST37 in β_1/2_-AR KO mice was not significantly different from that in WT littermates (unpaired *t*-test, n = 10 in each group). Deletion of M_2/3_-Rs significantly abolished the increase in phenol red excretion induced by acupuncture at ST37 compared with that in WT littermates (** P < 0.01, unpaired *t*-test, n = 10 in each group). **(F)** β_1/2_-AR knockout significantly diminished the decrease in phenol red excretion induced by acupuncture at ST25 relative to WT littermates (** P < 0.01, unpaired *t*-test, n = 10 in each group); the change in phenol red excretion induced by acupuncture at ST25 in M_2/3_-R KO mice was significantly different from that in WT littermates (** P < 0.01, unpaired *t*-test, n = 10 in each group).

In order to investigate whether the regulation of gastrointestinal motility by acupuncture at heterotopic or homotopic acupoints shapes gastrointestinal transition in β_1/2_-AR KO mice and M_2/3_-R KO mice, we administered phenol red via oral gavage to examine fecal residue. As shown in Fig 6A and 6B, basal phenol red excretion (non-MA) was slightly higher in β_1/2_-AR KO mice than in their WT littermates; however, no significant difference was observed (KO: 5.02 ± 1.32 mg/L (Fig 6A), 4.93 ± 0.78mg/L (Fig 6B); WT: 4.29 ± 1.17 mg/L (Fig 6A), 4.38 ±1.08 mg/L (Fig 6B)); a significant increase in phenol red excretion was found in M_2/3_ knockout mice compared with that in WT (KO: 8.16 ± 1.21 mg/L and 7.69 ±2.07 mg/L, WT: 4.39 ± 0.97 mg/L and 4.38 ± 1.08 mg/L; P < 0.01) (Fig 6C and 6D).

Fig 6A and 6C show the effects of MA at ST37 on gastrointestinal transition in β_1/2_-AR KO mice, M_2/3_-R KO mice, and their WT littermates. MA at ST37 was associated with a significant increase in phenol red concentration in the feces of β_1/2_-AR KO mice (MA: 10.76 ± 2.26 mg/L, non-MA: 5.02 ± 1.32 mg/L; increase of 116.75 ± 20.47%, P <0.01) as well as in those of their WT littermates (MA: 7.86 ± 0.92 mg/L, non-MA: 4.29 ± 1.17 mg/L; increase of 90.85 ± 18.91%, P < 0.05). No statistical difference in ST37 acupuncture-induced phenol red concentration was found between the feces of β_1/2_-AR KO mice and those of their WT littermates (β_1/2_-AR KO: 116.75 ± 20.47%; WT: 90.85 ± 18.91%) (Fig 6E). MA at ST37 failed to induce a significant increase in phenol red excretion in the feces of M_2/3_-R KO mice (MA: 9.77 ± 1.74 mg/L, non-MA: 8.16 ± 1.21 mg/L, increase of 17.87 ± 7.59%). However, in the WT littermates of M_2/3_-R KO mice, acupuncture at ST37 significantly increased phenol red excretion in feces (MA: 7.79 ± 1.55 mg/L, non-MA: 4.39 ± 0.97 mg/L; increase of 76.85 ± 11.49%, P < 0.01). A significant difference in ST37 stimulation-induced change of phenol red excretion was found between M_2/3_-R KO mice and their WT littermates (KO: 17.87±7.59%, WT: 76.85±11.49%, P < 0.01) (Fig 6E). These data suggest that M_2_ and M_3_ receptors play a critical role in the enhancement of gastrointestinal transition by acupuncture at ST37.

Fig 6B and 6D show the effects of MA at ST25 on gastrointestinal transition in β_1/2_-AR KO mice, M_2/3_-R KO mice, and their WT littermates. MA at ST25 was associated with significantly increased phenol red concentration in the feces of β_1/2_-AR KO mice (MA: 12.33 ± 1.81 mg/L, non-MA: 4.93 ± 0.78mg/L; increase of 155.92 ± 27.11%; P < 0.01). In their WT littermates, the increase in phenol red excretion in feces following MA at ST25 was not significant (MA: 4.18 ±0.93 mg/L non-MA: 4.38 ±1.08 mg/L). A significant difference in ST25 stimulation-induced increase in phenol red in feces was found between β_1/2_-AR KO mice and their WT littermates (KO: 155.92 ± 27.11%, WT: -10.29 ± 2.85%; P < 0.01) (Fig 6F). MA at ST25 induced a significant decrease of fecal phenol red excretion in M_2/3_-R KO mice (MA: 4.22 ± 1.18 mg/L, non-MA: 7.69 ± 2.07 mg/L; decrease of 44.95 ± 14.98%, P < 0.05). However, in their WT littermates, MA at ST25 triggered a statistically insignificant increase in fecal phenol red excretion (MA: 4.65 ± 0.83 mg/L, non-MA: 4.08 ± 1.01 mg/L; increase of 13.35 ± 1.30%). Following acupuncture at ST25, a statistical difference in phenol red excretion was found between M_2/3_-R KO mice and their littermate WT mice (KO: -44.95 ± 14.98%, WT:13.35 ± 1.30%; P < 0.01) (Fig 6F). These data suggest that acupuncture at ST25 triggers an increase in gastrointestinal transition via β_1/2_-AR deletion and a decrease in gastrointestinal transition as a result of M_2/3_-R deletion.

## Discussion

In the present study, we investigated the phenotypic and functional differences in the pylorus, jejunum, and distal colon between M_2/3_ KO mice, β_1/2_ KO mice, and their wild-type littermates at basal conditions and in response to manual acupuncture stimulation at the acupoints ST37 and ST25. We firstly observed the basal gastrointestinal phenotypes and found no significant differences between M_2/3_-R KO mice, β_1/2_-AR KO mice, or their WT littermates in terms of intraluminal pressure in the pylorus, jejunum, and distal colon, reflecting a similar baseline in gastrointestinal motility. However, the fecal dry-wet ratio in M_2/3_-R KO mice and β_1/2_-AR KO mice was higher than that of their WT littermates, suggesting the role of adrenergic and cholinergic receptors in modulation of water absorption in the gastrointestinal tract.

To our knowledge, the present study is the first to report that knockout of the M_2/3_ receptor significantly inhibits ST37 acupuncture-induced enhancement in gastric, jejunal, and colonic motility. Additionally, we found that knockout of the β_1/2_ receptor significantly reduces ST25 acupuncture-induced inhibition of gastric and jejunal motility, but has no effect on colonic motility. Furthermore, acupuncture stimulation at ST25 significantly increases gastrointestinal transition in β_1/2_-AR KO mice, yet significantly decreases gastrointestinal transition in M_2/3_-R KO mice, without altering gastrointestinal transition rate in WT animals. Therefore, our study revealed that M_2_/M_3_ receptors are required for the sufficient enhancement of gastrointestinal motility in whole gastrointestinal transition induced by acupuncture at heterotopic acupoints, whereas β_1_/β_2_ receptors are needed for acupuncture at homotopic acupoints to slow down gastrointestinal motility associated with whole gastrointestinal transition.

Our previous studies have demonstrated that manual acupuncture at acupoints innervated by different distant spinal segments to gastrointestinal innervations is capable of inducing facilitative effects on gastrointestinal motility. Conversely, acupuncture at various acupoints innervated by the same and adjacent spinal segments to gastrointestinal innervations induces an inhibitory effect on gastrointestinal motility [26]. The afferents of ST37 terminate at the spinal segment L5, which is heterotopic to gastrointestinal innervation. ST25 enters spinal segment T10, which has homotopic innervation to the stomach (T6-10) and jejunum (T9-12), but heterotopic innervation to the distal colon (L1-2), as the sacral parasympathetic efferent which innervates the distal colon mainly originates from the S1 spinal segment in rats [27]. Therefore, in the current study, ST37 represents a heterotopic acupoint to the stomach, jejunum, and distal colon, and ST25 a homotopic acupoint to the stomach and jejunum, but a heterotopic acupoint to the distal colon.

Sympathetic nerves that innervate the stomach originate from the lateral horn of spinal segments T9-T12. These preganglionic neurons project to postganglionic neurons in the celiac ganglia, from which noradrenergic postganglionic fibers originate and terminate at the enteric nervous system, or on gastric smooth muscles, to inhibit gastric motility via postsynaptic β receptor or presynaptic α_2_ receptor activation [28–31]. Previous studies have established that β_1_ and β_2_ receptor subtypes, which are predominantly expressed in the gastrointestinal tract, play an important role in mediating the inhibitory effects of the sympathetic nervous system on gastrointestinal motility [32, 33]. The inhibitory effect of acupuncture at abdominal acupoints may be blocked by guanidine or propranolol, suggesting that β-ARs play a critical role in mediating the acupuncture-induced inhibition of gastric motility [34]. However, it is still not clear which β receptor subtypes are involved in the regulation of gastric motility by acupuncture. In this study, acupuncture at ST25, the acupoint of homo-segmental innervations in both the stomach and jejunum, inhibited either gastric or jejunal motility in WT mice, consistent with previous reports [35, 25]. However, in β_1/2_-AR KO mice, the inhibitory effect induced by acupuncture at ST25 was significantly reduced compared to that observed in their WT littermates. Therefore, our findings indicate that β_1_ and β_2_ receptors play a critical role in mediating inhibitory gastric and jejunal motility induced by acupuncture at homotopic acupoints. These results are consistent with those of previous studies in which guanidine or propranolol was shown to block the effect of acupuncture [34]. Interestingly, ST25 also exhibited a hallmark signature of heterotopic acupoints in regulating distal colonic motility in β_1/2_-AR KO mice, as the increase in both intracolonic pressure and frequency, triggered by acupuncture at ST25, was not significantly different from that in WT mice. These results indicate that β_1_ and/or β_2_ receptors do not mediate the regulation of distal colonic motility by acupuncture at heterotopic acupoints. In particular, the data demonstrating that β_1/2_-AR deletion did not completely abolish ST25 acupuncture-induced inhibition of gastric and jejunal motility suggest that β_3_-AR is likely involved in mediating these effects, as β_3_-ARs were still expressed in the gastrointestinal tissues of β_1/2_-AR KO mice [11].

Parasympathetic nerves play a critical role in the excitatory regulation of gastrointestinal motility via acupuncture or acupuncture-like stimulation at heterotopic acupoints such as ST36 (Zusanli), ST37 (Shangjuxu), and LI11 (Quchi) [9, 36, 37]. It has been demonstrated that parasympathetic terminals release the contractile neurotransmitter acetylcholine (ACh). ACh exerts excitatory effects on smooth muscle tissues by binding to muscarinic receptors [38]. Muscarinic acetylcholine receptors, which are comprised of five distinct subtypes (M_1-5_) and widely distributed in smooth muscle tissue throughout the body (including in the gastrointestinal tract), mediate diverse autonomic functions [12–14, 39–43]; however, in gastrointestinal smooth muscles, the M_2_ and M_3_ muscarinic receptor subtypes are preferentially expressed [44]. ACh regulates gastrointestinal motility via the M_2_ and M_3_ receptors [45]. It is well documented that M_**3**_ receptors play a role in mediating regulation of the stomach, small intestine, and colon by ACh, and M_**3**_ receptor antagonists inhibit gastric motility and emptying [46, 47]. Although the M_2_ receptor is expressed in greater abundance than the M_3_ receptor in gastrointestinal smooth muscle tissue, this receptor is less active in terms of mediating cholinergic contractions in WT tissues. In addition, the M_2_ receptor directly mediates the residual contraction of M_3_-homozygous gastrointestinal muscles [48]. Both M_3_ and M_2_ receptors mediate contractions induced by stimulation of cholinergic nerves, and play a direct role in inducing contraction in gastric and ileal smooth muscles [9, 15–19]. The simultaneous activation of both M_2_ and M_3_ receptors is expected to elicit a greater contractile force than that generated under similar conditions when only M_3_ receptors are activated [49]. The significant inhibition of ST37 acupuncture-induced enhancement of gastric, jejunal, and colonic motility, as a result of M_2/3_ receptor knockout, significantly revealed that M_2_ and M_3_ receptors play a critical role in mediating gastrointestinal motility facilitated by acupuncture at heterotopic acupoints in mice. This finding was consistent with that of our previous study in which pharmacological approaches were utilized [9, 35]. It should be noted that, in M_2_/_3_-R KO mice, acupuncture at ST25 exerted differential effects on gastric and jejunal motility as well as distal colonic motility when simultaneous recordings were performed. In M_2/3_-R KO mice, acupuncture at ST25 not only decreased intragastric pressure and contraction frequency (no significant difference was observed relative to WT mice), but also reduced both intrajejunal pressure and contraction frequency (no significant difference was found relative to WT mice); however, acupuncture at ST25 was found to induce an increase in intracolonic pressure. These phenotypes are attributed to the hallmark homotopic connection of ST25 to the stomach and jejunum, and its heterotopic link to the distal colon. In particular, the increase in distal colonic motility induced by acupuncture at ST25 in M_2/3_-R KO mice was significantly less than in WT mice. This suggests that M_2/3_-R s additionally mediate the increase in motility in the distal colon following acupuncture at ST25. β_1/2_-ARs or M_2/3_-Rs expressed on gastrointestinal smooth muscles may be activated through multiple synapse-relayed neural pathways associated with sympathetic reflex (homotopic acupoints) or parasympathetic reflex (heterotopic acupoints). In this process, homotopic acupoint-induced signals may be relayed via fewer synapses, while heterotopic-acupoint-induced signals require a larger number of synapse relays [50–52]. Therefore, the differential gastrointestinal motility responses observed in this study may additionally reflect differences in multiple synapse-relayed neural pathways that transmit homotopic and heterotopic signals.

Gastroparesis, constipation, irritable bowel syndrome, and functional dyspepsia are human diseases associated with maladaptive alterations in gastrointestinal transit of food, chyme, and residue. Specific regions of the gastrointestinal tract (e.g., stomach, small intestine, or colon) play different roles in food transition. Based on the aforementioned acupuncture-triggered inhibition or stimulation of different gastrointestinal segments, acupuncture may promote or decrease food transition. In this study, acupuncture at ST37 increased gastric, jejunal, and distal colonic motility in β_1/2_-AR KO mice, thus increasing the concentration of phenol red in the feces, with no significant differences observed relative to WT littermates. However, in M_2/3_-R KO mice, acupuncture at ST37 increased the concentration of phenol red in the feces; however, this increase was smaller than that observed in WT mice. This observation was consistent with the finding that gastric, jejunal, and distal colonic motility were dramatically increased by acupuncture at ST37; however, this effect was blunted compared to that observed in the WT littermates. In β_1/2_-AR KO mice, acupuncture at ST25 significantly reduced the inhibition of gastric and jejunal motility, but did not affect distal colonic motility, thereby increasing fecal phenol red concentration. The observed increase was significantly larger than that observed in their WT littermates. In M_2/3_-R KO mice, acupuncture at ST25 significantly reduced fecal phenol red concentration relative to their WT counterparts, as the absence of M_2_ and M_3_ receptors significantly inhibited gastric and jejunal motility as well as inhibited the increase in distal colonic motility. As ST25 is a homotopic acupoint to the stomach and jejunum and a heterotopic acupoint to the distal colon, acupuncture at ST25 did not alter phenol red discharge in feces significantly in WT mice (Fig 6B and 6D). The jejunum and colon play a more influential role than the stomach in promoting food transition. Therefore, M_2_ and/or M_3_ receptors are required for motilities of different gastrointestinal segments facilitated by acupuncture at heterotopic acupoints, and deletion of M_2_ and/or M_3_ receptors inhibits the effect of acupuncture on motility and transition in the whole gastrointestinal tract. β_1_ and/or β_2_ receptors are required for decelerating motilities of most gastrointestinal segments by acupuncture at homotopic acupoints, and transition in the whole gastrointestinal tract is thus increased following β_1_ and/or β_2_ receptor deletion. Our findings additionally suggest that, in the treatment of gastrointestinal disorders, homotopic acupoints on the abdomen should be selected according to the specific region involved. In future studies, we will not only continue to focus on the roles of each β receptor subtypes or M receptor subtypes in gastrointestinal motility, secretion regulated by acupuncture at homotopic or heterotopic acupoints, but also investigate whether and how other specific receptors like calcitonin gene-related peptide (CGRP) receptors, 5-HT receptors mediate the different response of gastrointestinal motility, secretion induced by acupuncture at heterotopic or homotopic acupoints.

In summary, M_2_/M_3_ receptors are required for increasing gastrointestinal motility and accelerating food transition by acupuncture at heterotopic acupoints located on the limbs. β_1_ β_2_ receptors are needed for acupuncture at homotopic acupoints located on the abdomen to inhibit gastric motility and jejunal motility. The opposing effects induced by acupuncture at homotopic acupoints on different regions of the gastrointestinal tract may slow down gastric emptying and food transition, with clinical implications for the treatment of diseases involving gastrointestinal dysregulation.

## Supporting Information

S1 Fig Effect of Sham acupuncture at ST37 or ST25 on gastrointestinal motility. (A) Representative traces of gastric, jejunal, and distal colonic motility induced by sham or real acupuncture at ST25 in wildtype mice. (B) Representative traces of gastric, jejunal, and distal colonic motility induced by sham or real acupuncture at ST37 in wildtype mice. (C) Sham acupuncture at ST25 did not significantly decrease gastric motility, significant difference compared with real acupuncture at same acupoint (** P<0.01); sham acupuncture at ST37 did not significantly increase gastric motility, compared with real acupuncture at the same acupoint (* P<0.05). (D) Sham acupuncture at ST25 did not significantly inhibit jejunal motility, compared with real acupuncture at the same acupoint (** P<0.01); sham acupuncture at ST37 did not remarkably increase jejunal motility, significant difference compared with real acupuncture at same acupoint (* P<0.05). (E) Sham acupuncture at ST25 did not statistically enhance distal colonic motility, significant difference compared with real acupuncture at the same acupoint (** P<0.01); sham acupuncture at ST37 did not obviously increase distal colonic motility, significant difference compared with real acupuncture at the same acupoint (** P<0.01). Unpaired t test was applied, n = 5.
(TIF)

S1 File Genetic background of transgenic mice. (DOCX)

S2 File Effect of Sham acupuncture on gastrointestinal motility in wildtype mice. (DOCX)
