# Supplementary figures and images for: β1/2 or M2/3 Receptors Are Required for Different Gastrointestinal Motility Responses Induced by Acupuncture at Heterotopic or Homotopic Acupoints
Source: PLoS One. 2016 Dec 15;11(12):e0168200. doi: 10.1371/journal.pone.0168200 (PMC5158317; doi:10.1371/journal.pone.0168200)

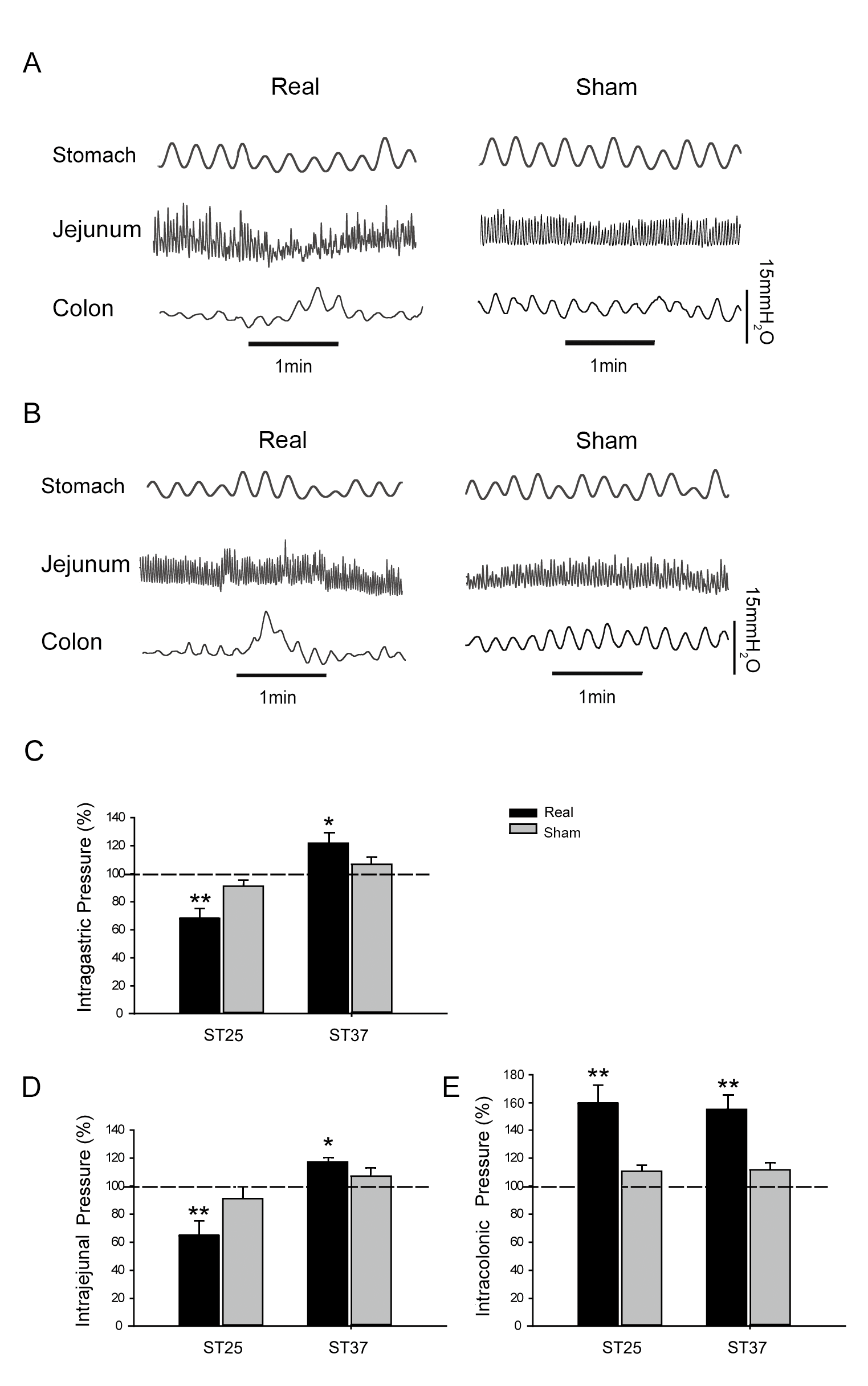

Supplement: S1 Fig — (A) Representative traces of gastric, jejunal, and distal colonic motility induced by sham or real acupuncture at ST25 in wildtype mice. (B) Representative traces of gastric, jejunal, and distal colonic motility induced by sham or real acupuncture at ST37 in wildtype mice. (C) Sham acupuncture at ST25 did not significantly decrease gastric motility, significant difference compared with real acupuncture at same acupoint (** P<0.01); sham acupuncture at ST37 did not significantly increase gastric motility, compared with real acupuncture at the same acupoint (* P<0.05). (D) Sham acupuncture at ST25 did not significantly inhibit jejunal motility, compared with real acupuncture at the same acupoint (** P<0.01); sham acupuncture at ST37 did not remarkably increase jejunal motility, significant difference compared with real acupuncture at same acupoint (* P<0.05). (E) Sham acupuncture at ST25 did not statistically enhance distal colonic motility, significant difference compared with real acupuncture at the same acupoint (** P<0.01); sham acupuncture at ST37 did not obviously increase distal colonic motility, significant difference compared with real acupuncture at the same acupoint (** P<0.01). Unpaired t test was applied, n = 5. (TIF) [file pone.0168200.s001.tif]
