# Supplementary material for: β1/2 or M2/3 Receptors Are Required for Different Gastrointestinal Motility Responses Induced by Acupuncture at Heterotopic or Homotopic Acupoints
Source: PLoS One. 2016 Dec 15;11(12):e0168200. doi: 10.1371/journal.pone.0168200 (PMC5158317; doi:10.1371/journal.pone.0168200)
Supplement: S1 File — (DOCX) [file pone.0168200.s002.docx]

**Genetic background of transgenic mice**

*β_1/2_^-/-^ mice*

As provided by Jackson laboratory [1], double β_1/2_ adrenergic receptor knockout mice were created by mating Adrb1 homozygous knockout mice with Adrb2 homozygous knockout mice to generate compound heterozygotes, the offspring of which were then mated to obtain compound homozygotes.

Adrb1 null mice were created using a targeting vector containing a neomycin resistance gene driven by the mouse phosphoglycerate kinase promoter to disrupt most of the Adrb1 coding region (all but a 3' 153 bp). The construct was transfected into 129- derived R1 embryonic stem (ES) cells. Correctly targeted ES cells were injected into C57BL/6J blastocysts. The resulting chimeric male animals were mated to C57BL/6J X DBA/2 F1 hybrids.

Adrb2 null mice were created in a similar fashion using a targeting vector again containing a neomycin resistance gene driven by the mouse phosphoglycerate kinase promoter to disrupt Adrb2 such that the end of the fourth transmembrane segment is absent, rendering the receptor nonfunctional. The construct was transfected into 129- derived R1 embryonic stem (ES) cells. Correctly targeted ES cells were injected into CD-1 blastocysts. The resulting chimeric male animals were mated to FVB/N females.

*M_2/3_^-/-^ mice*

M_2/3_ receptors knockout mice were required from Nanjing Biomedical Research Institute of Nanjing University. To generate animals deficient in both the M_2_ and M_3_ subtypes, homozygous mAChR2^-/-^mice were crossed with homozygous mAChR3^-/-^mice. The resulting F1 compound heterozygotes were then intercrossed to generate F2 mice. mAChR2/3^-/-^ mice were obtained at the expected Mendelian ratio and were subsequently interbred to generate the animals used for the experiments described here. F2 wild-type mice were interbred to obtain control mice (mAChR2/3^+/+^) with an equivalent genetic background [129/J1 (25%) X 129SvEv (25%) X CF1 (50%)] [1-5].

The M_2_ receptors knockout mice (mAChR2^-/-^ mice) and the corresponding wild-type mice (mAChR2^+/+^ mice) had 129J1 (50%) X CF1 (50%). The M_3_ receptors knockout mice (mAChR3^-/-^) and the corresponding wild-type mice (mAChR^+/+^ mice) 129SvEv (50%) X CF1 (50%). ^6^

**References**

1. <https://www.jax.org/strain/003810>
2. Gomeza J, Shannon H, Kostenis E, Felder C, Zhang L, Brodkin J, Grinberg A, Sheng H, and Wess J. Pronounced pharmacologic deficits in M2 muscarinic acetylcholine receptor knockout mice.Proc Natl Acad Sci. 1999;96:1692–1697.
3. Miyakawa T, Yamada M, Duttaroy A, and Wess J. Hyperactivity and intact hippocampus-dependent learning in mice lacking the M1 muscarinic acetylcholine receptor. J Neurosci. 2001; 21:5239–5250.
4. Yamada M, Miyakawa T, Duttaroy A, Yamanaka A, Moriguchi T, Makita R, Ogawa M, Chou CJ, Xia B, Crawley JN, et al. (2001) Mice lacking the M3 muscarinic acetylcholine receptor are hypophagic and lean. Nature. 2001;410:207–212.
5. Fisahn A, Yamada M, Duttaroy A, Gan JW, Deng CX, McBain CJ, and Wess J. Muscarinic induction of hippocampal gamma oscillations requires coupling of the M1 receptor to two mixed cation currents. Neuron. 2002;33:615–624.
6. [Struckmann N](https://www.ncbi.nlm.nih.gov/pubmed/?term=Struckmann%20N%5BAuthor%5D&cauthor=true&cauthor_uid=14645675), [Schwering S](https://www.ncbi.nlm.nih.gov/pubmed/?term=Schwering%20S%5BAuthor%5D&cauthor=true&cauthor_uid=14645675), [Wiegand S](https://www.ncbi.nlm.nih.gov/pubmed/?term=Wiegand%20S%5BAuthor%5D&cauthor=true&cauthor_uid=14645675), [Gschnell A](https://www.ncbi.nlm.nih.gov/pubmed/?term=Gschnell%20A%5BAuthor%5D&cauthor=true&cauthor_uid=14645675), [Yamada M](https://www.ncbi.nlm.nih.gov/pubmed/?term=Yamada%20M%5BAuthor%5D&cauthor=true&cauthor_uid=14645675), [Kummer W](https://www.ncbi.nlm.nih.gov/pubmed/?term=Kummer%20W%5BAuthor%5D&cauthor=true&cauthor_uid=14645675), [Wess J](https://www.ncbi.nlm.nih.gov/pubmed/?term=Wess%20J%5BAuthor%5D&cauthor=true&cauthor_uid=14645675), [Haberberger RV](https://www.ncbi.nlm.nih.gov/pubmed/?term=Haberberger%20RV%5BAuthor%5D&cauthor=true&cauthor_uid=14645675). Role of muscarinic receptor subtypes in the constriction of peripheral airways: studies on receptor-deficient mice. [Mol Pharmacol.](https://www.ncbi.nlm.nih.gov/pubmed/?term=Role+of+Muscarinic+Receptor+Subtypes+in+the+Constriction+of+Peripheral+Airways%3A+Studies+on+Receptor-Deficient+Mice) 2003 Dec;64:1444-1451.
