# Supplementary material for: β1/2 or M2/3 Receptors Are Required for Different Gastrointestinal Motility Responses Induced by Acupuncture at Heterotopic or Homotopic Acupoints
Source: PLoS One. 2016 Dec 15;11(12):e0168200. doi: 10.1371/journal.pone.0168200 (PMC5158317; doi:10.1371/journal.pone.0168200)
Supplement: S2 File — (DOCX) [file pone.0168200.s003.docx]

**Effect of Sham acupuncture on gastrointestinal motility in wildtype mice**

In this experiment, 5 C57/BL6 mice were used in each group. Sham acupuncture was performed with a little modification based on the previous reports [1, 2]. In brief, the needles were just inserted subcutaneously into the skin and not rotated in this sham acupuncture. As shown in the S1Fig A and C, in ST25, sham acupuncture reduced 11.42 ± 1.48 of baseline gastric pressure, with a significant difference compared to real acupuncture (31.57 ± 4.22, P<0.01, unpaired t test, n=5); in ST37, sham acupuncture increased 6.38 ± 2.77, significant difference compared with real acupuncture (22.59 ± 4.15, P<0.05, unpaired t test, n=5) (S1Fig B and C). As shown in S1Fig A and D, sham acupuncture at ST25 decreased 11.12 ± 5.13 of baseline jejunal pressure, significant difference compared with real acupuncture at ST 25 (37.21 ± 6.71, P<0.01, unpaired t test, n=5); S1Fig B and D showed that sham acupuncture at ST37 increased 7.18 ± 3.23, significant difference compared with real acupuncture at ST37 (18.22 ± 1.94, P<0.05, unpaired t test, n=5). As shown in S1Fig A and E, in ST25, either sham and real acupuncture increased colonic pressure but with a significant difference between groups (sham vs real: 11.55 ± 1.88 vs 60.51 ±7.16, P<0.01, unpaired t test, n=5); as shown in S1Fig B and E, in ST37, sham acupuncture induced an increase of 13.25 ± 3.81, significant difference compared with real acupuncture (57.43 ± 5.18, P<0.01, unpaired t test, n=5).

**References**

1. Li G, Huang C, Zhang X, Xie H, Cheng H, Tang Y, Li Z. The short-term effects of acupuncture on patients with diabetic gastroparesis: a randomised crossover study. Acupunct Med. 2015 Jun;33(3):204-9.
2. Liu H, Zhang Y, Qi D, Li W. Downregulation of the spinal NMDA receptor NR2B subunit during electro-acupuncture relief of chronic visceral hyperalgesia. J Physiol Sci. 2016 May 25. PMID: 27221284
